# Long-Term Oil Contamination Alters the Molecular Ecological Networks of Soil Microbial Functional Genes

**DOI:** 10.3389/fmicb.2016.00060

**Published:** 2016-02-03

**Authors:** Yuting Liang, Huihui Zhao, Ye Deng, Jizhong Zhou, Guanghe Li, Bo Sun

**Affiliations:** ^1^State Key Laboratory of Soil and Sustainable Agriculture, Institute of Soil Science, Chinese Academy of SciencesNanjing, China; ^2^State Key Joint Laboratory of Environment Simulation and Pollution Control, School of Environment, Tsinghua UniversityBeijing, China; ^3^Key Laboratory of Environmental Biotechnology, Research Center for Eco-Environmental Sciences, Chinese Academy of SciencesBeijing, China; ^4^Department of Botany and Microbiology, Institute for Environmental Genomics, University of Oklahoma, NormanOK, USA

**Keywords:** microbial interaction, oil contamination, molecular ecological network, functional genes, hydrocarbon degradation

## Abstract

With knowledge on microbial composition and diversity, investigation of within-community interactions is a further step to elucidate microbial ecological functions, such as the biodegradation of hazardous contaminants. In this work, microbial functional molecular ecological networks were studied in both contaminated and uncontaminated soils to determine the possible influences of oil contamination on microbial interactions and potential functions. Soil samples were obtained from an oil-exploring site located in South China, and the microbial functional genes were analyzed with GeoChip, a high-throughput functional microarray. By building random networks based on null model, we demonstrated that overall network structures and properties were significantly different between contaminated and uncontaminated soils (*P* < 0.001). Network connectivity, module numbers, and modularity were all reduced with contamination. Moreover, the topological roles of the genes (module hub and connectors) were altered with oil contamination. Subnetworks of genes involved in alkane and polycyclic aromatic hydrocarbon degradation were also constructed. Negative co-occurrence patterns prevailed among functional genes, thereby indicating probable competition relationships. The potential “keystone” genes, defined as either “hubs” or genes with highest connectivities in the network, were further identified. The network constructed in this study predicted the potential effects of anthropogenic contamination on microbial community co-occurrence interactions.

## Introduction

Increased exploration and exploitation of oil resources have resulted in severe contamination worldwide (Kvenvolden and Cooper, 2003). Hundreds of micrograms of oil per gram of soil were detected in several heavily contaminated sites (Liang et al., 2012). Oil contamination alters the biotic taxonomic composition and physical and chemical properties of an environment, thus posing considerable threat to the ecological systems (Kingston, 2002). Oil contamination considerably affects the structures and functional diversity of microbial communities, including bacteria, fungi, and archaea (Liang et al., 2011; Lu et al., 2012; Bell et al., 2014). The overall microbial population diversity in oil-contaminated sites usually declines (Van Hamme et al., 2003), particularly, of those microbes participating in carbon and nitrogen cycling. The loss of microbial diversity and changes in community composition alter their functional processes. Although soil respiration can be stimulated as an enrichment of the biodegradable carbon source after new oil spills, an inhibitory effect on the hydrolase activities involved in nitrogen, phosphorus, or carbon cycles is observed (Labud et al., 2007). Although many microbial species can degrade oil contaminants (Atlas, 1981; Das and Chandran, 2011), effective oil removal is critically dependent on community-level functions. For example, the biodegradation of refractory components of oil contaminants, such as high molecular-weight polycyclic aromatic hydrocarbons (PAHs), requires the co-metabolism of several species (Nzila, 2013).

The microbial functional genes involved in the degradation of specific compounds in hydrocarbon-contaminated environments can be used as indicators of the biodegradation potential of the corresponding compounds and their bioavailability and transport in the environment (Stapleton and Sayler, 2000; Marlowe et al., 2002; Mesarch et al., 2004; Tuomi et al., 2004). The abundance of contaminant-degrading genes highly correlates with the concentrations of contaminant, as well as the efficiency at which the hydrocarbons are mineralized (Fleming et al., 1993; Park and Crowley, 2006; Salminen et al., 2008). In some PAH-contaminated soils and sediments, the abundance or expression of naphthalene-degrading genes is correlated with naphthalene concentrations (Dionisi et al., 2004; Cébron et al., 2008). Functional genes are also useful in monitoring the dynamics of contaminant-degrading bacteria in microcosms (Ringelberg et al., 2001; Sei et al., 2003) and evaluating hydrocarbon biodegradability (Cavalca et al., 2004; Baldwin et al., 2008).

The rapid methodological development of high-throughput metagenomic sequencing (Simon and Daniel, 2011) and microarray techniques (He et al., 2012) has dramatically advanced our understanding of the diverse and complex microbial functional communities in recent years. The sequencing technology uses Roche 454 or Illumina platforms to capture sequences for both targeted genes with available primers and metagenomics. As such, the approach provides novel insights into the phylogenetic and functional diversity as well as structure and composition of microbial communities. Shotgun metagenomic and metatranscriptomic sequencing revealed that genes for aliphatic hydrocarbon degradation are significantly enriched and expressed in hydrocarbon plume samples compared with uncontaminated seawater (Mason et al., 2012). Penton et al. (2013) evaluated new primers that target the dioxin- and dibenzofuran- degrading genes *dxnA1*, *dbfA1*, and *carAa*, found that the majority of the obtained environmental sequences were classified into novel sequence clusters in polychlorinated biphenyl-contaminated rhizosphere soil. However, in gene-targeted metagenomics analysis, each unique gene requires individual considerations in terms of primer design and sequence processing and classification. Microarray techniques, such as GeoChip, provide another approach for profiling the functional composition of known microbial populations by targeting hundreds to thousands of different gene families that play important roles in various biogeochemical processes at a time (He et al., 2010; Tu et al., 2014). In comparison with genome-resolved-metagenomic shotgun sequencing, GeoChip is required for primer matches that previously unsequenced organisms with divergent gene sequences will not be detected, and connection of organism with function is less clear. Nevertheless, GeoChip avoids the oversampling of dominant populations and is less challenging to employ in sequence assembly and data analysis than other approaches (Zhou et al., 2015). GeoChip 3.0 contains approximately 28000 50-mer oligonucleotide probes specific to the target genes, covering 292 functional gene families involved in carbon, nitrogen, phosphorus, and sulfur cycles, as well as energy metabolism, antibiotic resistance, metal resistance, and organic contaminant degradation (He et al., 2010). For organic contaminant degradation, a gene from each step of a contaminant degradation pathway is selected for probe design to monitor various degradation pathways. A total of 173 genes/enzymes are selected to detect the potential for degradation of 86 organic contaminants commonly found in the environment. These genes/enzymes mainly include 38 enzymes involved in the aromatic carboxylic acid (for example, benzoate, phenylpropionate, and phthalate) degradation, 18 for BTEX (benzene, toluene, ethylbenzene, and xylene), 10 for chlorinated aromatics (for example, 2-, 3-, and 4-chlorobenzoate, 2,4,5-trichlorophenoxyacetic acid), nine for heterocyclic aromatics (for example, carbazole and dibenzothiophene), nine for nitroaromatics (for example, nitrobenzene and nitrophenol), 18 for polycyclic aromatics (for example, biphenyl, fluorene, and naphthalene), 22 for other aromatics (for example, aniline, catechol, phenol), 15 for other hydrocarbons (for example, alkanes, cyclohexane, and tetrahydrofuran; He et al., 2010). The organic contaminant degradation gene probes on GeoChip are usually derived from the genes with known biological functions and microbial populations. Therefore, linking microbial diversity to ecosystem processes and functions is easily achieved in oil-contaminated sites through GeoChip. Several studies indicated that oil contamination significantly changes the composition and diversity of microbial functional genes by using GeoChip (Yergeau et al., 2007; Hazen et al., 2010; Liang et al., 2011; Lu et al., 2012). In our recent study on microbial functional gene diversity across five oil-contaminated sites, long-term oil contamination significantly decreases microbial alpha- (gene number, richness and the Shannon index) and beta-diversity (distance decay relationship; Liang et al., 2015). Moreover, the diversity changes along with the increase in deterministic assembly processes in soil microbes. However, little could be inferred quantitatively on the interactions among different microbial species/populations, which is of critical importance in maintaining the stability and functions of the community.

Microbial communities are highly complex, diverse, evolving systems. Therefore, the behavior and characteristics of these communities are difficult to predict compared with macro-ecological systems. Beyond basic survey on microbial community composition and diversity, investigating large environmental datasets to determine potential interactions between microbial species and functions remains a challenge (Raes et al., 2007). Barberan et al. (2012) assumed that microbial relationships can be depicted under the following principle. When two species (or any operational taxonomic units) co-occur or show similar abundance patterns over multiple samples, a positive relationship is assumed (Faust and Raes, 2012). Conversely, a negative relationship is considered when two species show mutual exclusion or anticorrelation (Faust and Raes, 2012). Given the criteria, a novel random matrix theory (RMT)-based network approach has been recently developed to delineate and characterize functional molecular ecological networks (fMENs) that involve microbial functional genes (Zhou et al., 2010). This approach provides a robust method to decipher the potential interactions of complex microbial communities. A general framework for fMENs is as follows (Zhou et al., 2010): (i) functional gene abundance data are collected on array hybridization; (ii) a pairwise Pearson correlation between any two genes is estimated on the basis of gene abundance data, and the absolute values of pairwise correlation coefficients are used to measure similarities; (iii) the similarity matrix is then transformed into an adjacency matrix by applying a threshold to the correlation values based on an RMT approach, which measures the strengths of connections between nodes; (iv) the strengths of the connections of each gene with all of the other connected genes are summed, yielding a connectivity of a single network. The connectivity represents the strength of the connection of a gene to all of the other genes in the network. In this network, a node represents a functional gene. Meanwhile, the edge linking two nodes represents the relationship between these two genes or potential functions (positive or negative). The edge weights represent the relationship strength, whereas the node size represents the abundance of genes or the node properties. The ecological networks determined via this method should reflect the co-occurrence among different microbial populations carrying the functional genes of interest rather than the individual “species” in a microbial community. Currently, little is known about whether and how oil contamination changes interactions among different microbial functional groups. This alternation may be affected by the introduction of contamination because increasing disturbance promotes interspecies competition (Simon and Daniel, 2011).

In a previous study, we found that an significant increase (68%) in the deterministic selection processes that shape the community composition and structure in contaminated soils with respect to that in uncontaminated soils in the Baise site (an oil-exploring site located in South China), which is highest than those in all the other sites (Liang et al., 2015), indicating that oil contamination may alter microbial assembly networks most in the Baise site. In this paper, we have re-analyzed the GeoChip data from the Baise site soils presented in Liang et al. (2015) using the fMEN approach in order to examine the interaction between specific groups of functional genes involved in carbon, nitrogen, phosphorus cycling, metal resistance, organic contaminant degradation. The following research questions were addressed: (i) whether oil contamination affects the functional network structure of soil microbial functional genes, (ii) what the potential “keystone genes” in the network are and how they change in response to contamination, and (iii) what the relationships are among genes functioning in the degradation of different oil components.

## Materials and Methods

### Sampling Sites and GeoChip Hybridization

Twenty soil samples were collected from Baise district (BS; 23°43′N, 107°04′E) in South China in May 2008 for network analyses of microbial functional genes. BS has a subtropical humid monsoon climate, with a mean annual rainfall of 1115 mm. Of these samples, 10 contaminated were collected from an oil-contaminated site in Baise Oilfield. Contaminated soils were collected adjacent to the crude oil pumping wells within a 2 km^2^ area where contamination occurred during oil extraction in 2004 according to record. The other 10 uncontaminated samples were taken simultaneously from undisturbed pristine soils about 5 km away from the contaminated site. At each sampling point, five soil cores (2.5 cm in diameter) within 0.015 m^2^ of the upper 10 cm soil were obtained and mixed thoroughly. The 20 soil samples were then individually used for microbial and chemical analyze. The collected soils were sealed in sterile sampling bags without air and transported to the laboratory on ice.

The oil contamination in BS oil field ranged from 12.1 mg/g to 168 mg/g. The contents of the four components of oil, namely, aliphatic hydrocarbons, aromatic hydrocarbons, polar fraction with heteroatoms of nitrogen, sulfur, and oxygen (NSO fraction), and asphaltenes, ranged from 28.7% to 53.6%, 8.9% to 25.7%, 10.0% to 23.9%, and 0.9% to 9.6%, respectively (Liang et al., 2012). The soil physical and chemical parameters in the contaminated soils were as follows: pH, 5.8 ± 0.52; water content, 17.3% ± 6.9%; total nitrogen (nitrogen in all organic and inorganic forms), 1176 ± 244 mg/kg; available nitrogen (NO_3_^-^, NO_2_^-^, and NH_4_^+^), 51.9 ± 30.3 mg/kg; total phosphorus (phosphorus in all organic and inorganic forms), 884 ± 403 mg/kg; available phosphorus (PO_4_^3-^), 22.9 ± 12.6 mg/kg; total organic carbon, 5.7% ± 7.4%; and soluble salts, 0.17% ± 0.07%. In comparison, the physical and chemical parameters in the uncontaminated soils were as follows: pH, 6.2 ± 0.59; water content, 20.1% ± 4.9%; total nitrogen, 952 ± 275 mg/kg; available nitrogen, 43.5 ± 25.1 mg/kg; total phosphorus, 854 ± 149 mg/kg; available phosphorus, 10.2 ± 13.1 mg/kg; total organic carbon, 3.4% ± 3.8%; and soluble salts, 0.06% ± 0.05% (Liang et al., 2012).

Biolog EcoPlate^TM^ (Biolog, Inc., Hayward, CA, USA), which contains three replicated wells of 31 carbon substrates, was used to investigate the carbon metabolic activity among the aerobic and heterotrophic bacterial communities in all the soil samples. Plates were incubated at room temperature (20°C). The optical density (λ = 590 nm) of each well was determined immediately (0 h) and every 24 h thereafter up to 180 h with a BioTek plate reader (ELX800; BioTek Inc., Winooski, VT, USA). Average well color development method (Garland and Mills, 1991) was used for Biolog data analysis.

Microbial genomic DNA was extracted from 20 soil samples individually. Particularly, 5 g of well-mixed soil from each sample was subjected to DNA extraction and purification (Zhou et al., 1996; Moore and Dowhan, 2002). An aliquot of 2 μg of DNA from each sample was directly labeled and hybridized with GeoChip 3.0 in triplicates as described previously (Liang et al., 2010, 2015). Microarray data processing was performed in the Microarray Data Manager system of the Institute for Environmental Genomics (IEG)^1^

### Network Construction and Characterization

To elucidate the effect of oil contamination on the overall microbial ecological network, we constructed two fMENs for the contaminated and uncontaminated soils, respectively. The GeoChip hybridization intensity data were log-transformed prior to the construction of a Pearson correlation matrix (Horvath and Dong, 2008) and converted to a similarity matrix. This similarity matrix measures the degree of concordance between the abundance profiles of genes across various samples by obtaining the absolute values of the correlation matrix (Horvath et al., 2006). Using RMT, ecological communities can be predicted by two universal extreme distributions of the nearest-neighbor spacing distribution of eigenvalues. These two distributions are the Gaussian orthogonal ensemble (GOE) statistics, which reflects the random properties of a complex system, and the Poisson distribution, which is related to the system-specific, non-random properties of a complex system (Luo et al., 2007). A threshold *s*_t_ can be defined as the transition of the nearest-neighbor spacing distribution of eigenvalues from GOE to Poisson distribution (Zhou et al., 2010). Subsequently, an adjacency matrix, which encodes the connection strength between each pair of nodes, was derived from the similarity matrix by applying the threshold (Luo et al., 2006, 2007). In this study, clear transitions of the nearest-neighbor spacing distribution of eigenvalues from GOE to Poisson distribution were observed for soil microbial communities with and without contamination. These transitions are indicated by the existence of a similarity threshold (**Table 1**). The topological indices of the network, such as the average degree (connectivity), average path length, and average clustering coefficient, were calculated to describe the properties of the two networks. The definitions and calculations of these indices were described previously (Zhou et al., 2010). Hartwell et al. (1999) defined a module in a biological system as “a discrete unit whose function is separable from those of other modules.” They suggested that the functional modules comprise a “critical level of biological organization.” In fMENs, a module is defined as a group of functional genes that are highly connected among themselves but exhibit few connections with the functional genes under other modules (Zhou et al., 2010). Modularity (M) measures the extent to which nodes attain more links within their own modules than expected if the linkage was random. The modularity was calculated as follows:

**Table 1:** Topological properties of the empirical molecular ecological networks (MENs) of microbial communities in uncontaminated (U) and contaminated (C) soils of the oil-contaminated site and their associated random MENs.

		Empirical networks								Random networks^d^		
			
Comm-unity	No. of original genes^a^	Similarity threshold (*s*_t_)	Network size (*n*)^b^	Links	*r* of scale free^c^ (significance)	Avg. connectivity (*avgK*)	Avg. path length (GD)^e^	Avg. clustering coefficient (*avgCC*)	Modularity (No. of modules)	Avg. path length (GD)	Avg. clustering coefficient (*avgCC*)	Modularity (M)
BS-U	3367	0.95	754	1150	–0.86 (*P* < 0.001)	3.05	0.907^f^	0.153^g^	0.79^h^ (141)	3.016 ± 0.044	0.162 ± 0.005	0.455 ± 0.012
BS-C	771	0.96	256	957	–0.88 (*P* < 0.001)	7.48	1.968^f^	0.292^g^	0.46^h^ (33)	2.826 ± 0.101	0.081 ± 0.008	0.233 ± 0.007


*^a^Number of genes that originally used for network construction using the RMT-based approach.*

*^b^Number of genes (i.e., nodes) in a network.*

*^c^Correlation coefficient (*r*) of the linear relationship in log[*P*(*k*)] - γlog(*k*), where *P*(*k*) is the fraction of connectivity *k* and γ is a constant.*

*^d^The random networks were generated by rewiring all of the links of a MEN with the identical numbers of nodes and links to the corresponding empirical MEN.*

*^e^GD, geodesic distance.*

*^f^Significant difference (*P* < 0.001) in average path length between contaminated and uncontaminated soils based on the Student *t-*test with standard deviations derived from corresponding random networks.*

*^g^Significant difference (*P* < 0.001) in average clustering coefficient between contaminated and uncontaminated soils based on the Student *t-*test with standard deviations derived from corresponding random networks.*

*^h^Significant difference (*P* < 0.001) in modularity between contaminated and uncontaminated soils based on the Student *t-*test with standard deviations derived from corresponding random networks.*

$$M=\overset{N_{M}}{\underset{s=1}{\Sigma }}\left[\frac{l_{s}}{L}-{\left(\frac{d_{s}}{2L}\right)}^{2}\right]$$

where *N*_M_ is the number of modules, *L* is the number of links in the network, *l*_s_ is the number of links between nodes in module *s*, and *d*_s_ is the sum of the degrees of nodes in module *s*. The module identification algorithm aims to determine the partition with largest modularity (Clauset et al., 2004). After scanning all branches of the hierarchical tree of a graph, the level with the maximum modularity score was used to separate the graph into multiple dense subgraphs (Clauset et al., 2004).

Each node can be assigned a role based on its topological properties. The role of node *i* is characterized by two parameters. The first parameter is within module connectivity (*Z*_i_), which describes how well a node is connected to the other nodes within its own module (Guimera and Amaral, 2005). *Zi* is described as follows:

$$Z_{i}=\frac{\kappa_{i}-{\overset{¯}{\kappa }}_{S_{i}}}{\sigma_{\kappa_{S_{i}}}}$$

where *κ_i_* is the number of links of node *i* to the other nodes within its module *s*_i_, ${\overset{¯}{\kappa }}_{s_{i}}$ is the average of *k* over all the nodes in *s_i_*, and σ_κ_s_ i___ is the standard deviation of κ in *s*_i_.

The second parameter is the connectivity among modules (*P*_i_), which reflects how well a node connects to various modules (Guimera and Amaral, 2005). *P*_i_ is given as follows:

$$P_{i}=1-\overset{N_{M}}{\underset{s=1}{\Sigma }}{\left(\frac{\kappa_{is}}{\kappa_{i}}\right)}^{2}$$

where *κ_is_* is the number of links of node *i* to nodes in module *s*, and *k_i_* is the total degree of node *i*.

Only one network was constructed by combining 10 samples under each condition; hence, we cannot statistically compare the network indices between contaminated samples and the control. Thus, random networks were generated to assess the statistical significance of network indices by using the Maslov and Sneppen (2002) procedure based on a null model. This model keeps the numbers of nodes and links unchanged but rewires all of the links’ positions in the “null model” network (Maslov and Sneppen, 2002). Consequently, the network sizes are the same, and the random rewired networks are comparable to the original ones. For each network identified in this study, 100 randomly rewired networks were generated, and all of the network indices were calculated individually. The average and standard deviation for each index of all of the random networks were obtained (Zhou et al., 2010). For comparison of the network indices under different conditions, the Student *t*-test was employed using the standard deviations derived from corresponding random networks.

All of the above calculations were carried out in the IEG website^2^ The present study is focused on the interactions of oil contaminant degradation-related functional genes. Thus, the fMENs were also constructed and visualized using the Cytoscape 2.6.0 [26] software for two functional categories, particularly, alkaline and PAH degradations.

### Relationships of Microbial Interaction Networks with Soil Variables

Gene significance was calculated on the basis of a sample geochemical trait (Zhou et al., 2010) across 10 replicate samples under oil contamination and non-contamination. Given that the measurement units for different soil variables vary, all of the trait data were standardized before statistical analysis. Correlations between the gene significance and the connectivity of individual genes were calculated. The statistical significances of these correlations were estimated on the basis of *P*-values.

## Results

### Effects of Oil Contamination on Microbial fMENs

fMENs were constructed for both contaminated and uncontaminated soils to determine the effect of oil contamination on microbial functional gene co-occurrence (**Table 1**). The network sizes, links, connectivities, and module numbers were calculated for microbial functional genes in the contaminated and uncontaminated soils. Random networks were generated to test the statistical significance of the network indices. Results indicated that the network indices, such as average path length, average clustering coefficient, and modularity, were significantly different between the contaminated and uncontaminated soils (*P* < 0.001). The overall network structures of the soil microbial communities were distinctly different under oil contamination.

### Visualization of the Topological Roles of Individual Nodes

**FIGURE 1:**
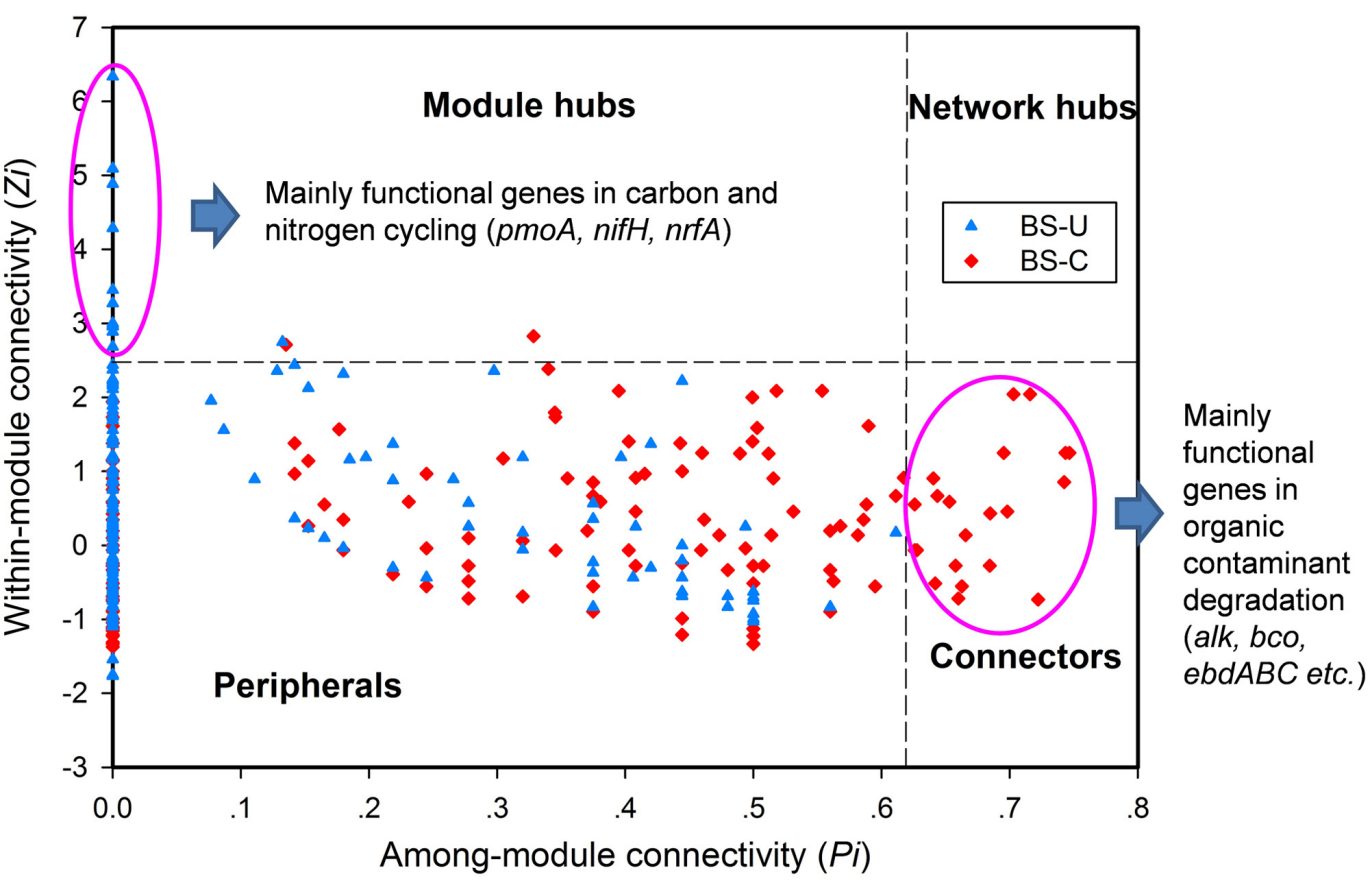
**Z–P plot showing the distribution of genes based on their topological roles from the BS oil-exploring site.** U, uncontaminated soils (blue), C, contaminated soils (red).

In a network developed from gene abundance data to represent the ecological co-occurrence (links) of different gene markers (nodes) in a microbial community, different nodes play distinct roles (Guimera et al., 2007; Fuhrman and Steele, 2008). Within-module connectivities (*Z*_i_) and among-module connectivities (*P*_i_) of both contaminated and uncontaminated soils were calculated and visualized in **Figure 1** to understand the effect of oil contamination on the topological roles of individual nodes. In this study, we followed the simplified classification as follows: (i) peripheral nodes (*Z*_i_ ≤ 2.5, *P*_i_ ≤ 0.62), which possess only a few links that are almost always to nodes within their modules; (ii) connectors (*Z*_i_ ≤ 2.5, *P*_i_ > 0.62), which are highly linked to several modules; (iii) module hubs (*Z*_i_ > 2.5, *P*_i_ ≤ 0.62), which are highly connected to numerous genes in their own modules; and (iv) network hubs (*Z*_i_ > 2.5, *P*_i_ > 0.62), which act as both module hubs and connectors. The threshold value of *Z*_i_ was determined by the density landscape of the nodes; a clear boundary at *Z*_i_ = 2.5 was observed, and *Z*_i_ > 2.5 was relatively “washed” down by the background of the non-hub region as described previously (Guimera and Amaral, 2005). Similarly, the *P*_i_ parameter space could be partitioned into different regions with a boundary at *P*_i_ = 0.62 by identifying the basins of attraction for the different node density plots (Guimera and Amaral, 2005). **Tables 2** and **3** provide detailed information on the module hub genes and connectors. The majority of the genes, particularly, about 98.4 and 90.2% of uncontaminated and contaminated soils, respectively, were peripherals, having most of their links inside their modules. Fewer module hub genes were present in contaminated soils (two genes) compared with the control (12 genes). By contrast, more genes playing as connectors were found in contaminated soils (23 genes), and none in the control. None of the module hub genes, and connectors was overlapped in the contaminated and uncontaminated samples. Furthermore, no network hub genes were noted in the two conditions.

**Table 2:** Information on the module hub genes.

	Gene ID	*Z*_i_	*P*_i_	Gene name	Gene category	Sub-category	Organism
bs-u	78221327	6.336	0.000	*cytochrome*	Energy process	Energy process	*Geobacter metallireducens GS-15*
	104304169	5.091	0.000	*pmoA*	Carbon cycling	Methane	Uncultured bacterium
	10863129	4.884	0.000	*nifH*	Nitrogen	Nitrogen fixation	*Lactate SRB-Enrichment culture clone HBLac1*
	46106	4.287	0.000	*nifH*	Nitrogen	Nitrogen fixation	*Rhodobacter capsulatus*
	146295293	3.451	0.000	*CadA*	Metal Resistance	Cadmium	*Caldicellulosiruptor saccharolyticus DSM 8903*
	18461102	3.270	0.000	*phenol_oxidase*	Carbon cycling	Carbon degradation	*Lentinula edodes*
	156719686	3.000	0.000	*mer*	Metal Resistance	Mercury	*Hydrogenobaculum* sp. *Y04AAS1*
	109645564	2.973	0.000	*nrfA*	Nitrogen	Dissimilatory N reduction	*Desulfitobacterium hafniense DCB-2*
	104304111	2.958	0.000	*pmoA*	Carbon cycling	Methane	Uncultured bacterium
	57903688	2.885	0.000	*gyrB*	Other category	Phylogenetic marker	*Xylella fastidiosa*
	28566177	2.749	0.133	*gyrB*	Other category	Phylogenetic marker	*Entomoplasma somnilux*
	103487991	2.682	0.000	*mer*	Metal Resistance	Mercury	*Sphingopyxis alaskensis RB2256*
							
bs-c	88789151	2.828	0.328	*MFS_antibiotic*	Antibiotic resistance	transporter	*Nitrococcus mobilis Nb-231*
	30248392	2.711	0.135	*czcA*	Metal Resistance	Cadmium, Cobalt, Zinc	*Nitrosomonas europaea ATCC 19718*


*ID, identification.*

**Table 3:** Information on the connector genes.

	Gene ID	*Z*_i_	*P*_i_	Gene name	Gene category	Sub-category	Organism
bs-u	none						
							
bs-c	6324893	1.250	0.747	*alkK*	Organic Remediation	Others	*Saccharomyces cerevisiae*
	62468076	1.250	0.743	*bco*	Organic Remediation	Aromatics	Uncultured bacterium
	33413585	0.854	0.743	*endochitinase*	Carbon cycling	Carbon degradation	*Trichoderma atroviride*
	88701127	-0.732	0.722	*mauAB*	Organic Remediation	Herbicide-related compound	*Congregibacter litoralis KT71*
	89512930	-0.732	0.722	*nifH*	Nitrogen	Nitrogen fixation	Uncultured nitrogen-fixing bacterium
	118705772	2.043	0.716	*mauAB*	Organic Remediation	Herbicide-related compound	*Sphingomonas wittichii RW1*
	67933455	2.043	0.703	*CODH*	Carbon cycling	Carbon fixation	*Solibacter usitatus Ellin6076*
	91802739	0.457	0.698	*czcA*	Metal Resistance	Cadmium, Cobalt, Zinc	*Nitrobacter hamburgensis X14*
	12659186	1.250	0.695	*nifH*	Nitrogen	Nitrogen fixation	*Treponema denticola*
	192808970	0.432	0.685	*Tet*	Antibiotic resistance	Others	*Geobacillus* sp. *Y412MC10*
	119963032	-0.276	0.684	*nmoA*	Organic Remediation	Aromatics	*Arthrobacter aurescens TC1*
	82724314	0.138	0.666	*endochitinase*	Carbon cycling	Carbon degradation	*Clostridium beijerinckii NCIMB 8052*
	133919284	-0.555	0.663	*B_lactamase_A*	Antibiotic resistance	Beta-lactamases	*Leminorella richardii*
	89075780	-0.719	0.660	*B_lactamase_A*	Antibiotic resistance	Beta-lactamases	*Photobacterium* sp. *SKA34*
	56476743	-0.276	0.658	*ebdABC*	Organic Remediation	Aromatics	*Azoarcus* sp. *EbN1*
	2196830	0.588	0.653	*gyrB*	other category	Phylogenetic marker	*Shewanella algae*
	157363044	0.668	0.644	*pcc*	Carbon cycling	Carbon fixation	*Thermotoga lettingae TMO*
	118685870	-0.515	0.642	*proO*	Organic Remediation	Aromatics	*Marinomonas* sp. *MWYL1*
	110647328	0.905	0.640	*czcA*	Metal Resistance	Cadmium, Cobalt, Zinc	*Alcanivorax borkumensis SK2*
	67920251	-0.065	0.628	*pcaG*	Organic Remediation	Aromatics	*Crocosphaera watsonii WH 8501*
	87135386	0.552	0.626	*mauAB*	Organic Remediation	Herbicides related compound	*Novosphingobium aromaticivorans DSM 12444*
	94554094	-0.065	0.626	*CopA*	Metal Resistance	Copper	*Deinococcus geothermalis DSM 11300*
	15806552	0.915	0.617	*pcc*	Carbon cycling	Carbon fixation	*Deinococcus radiodurans R1*


*ID, identification.*

### Network Interactions of Functional Genes Involved in Alkane and PAH Degradation

**FIGURE 2:**
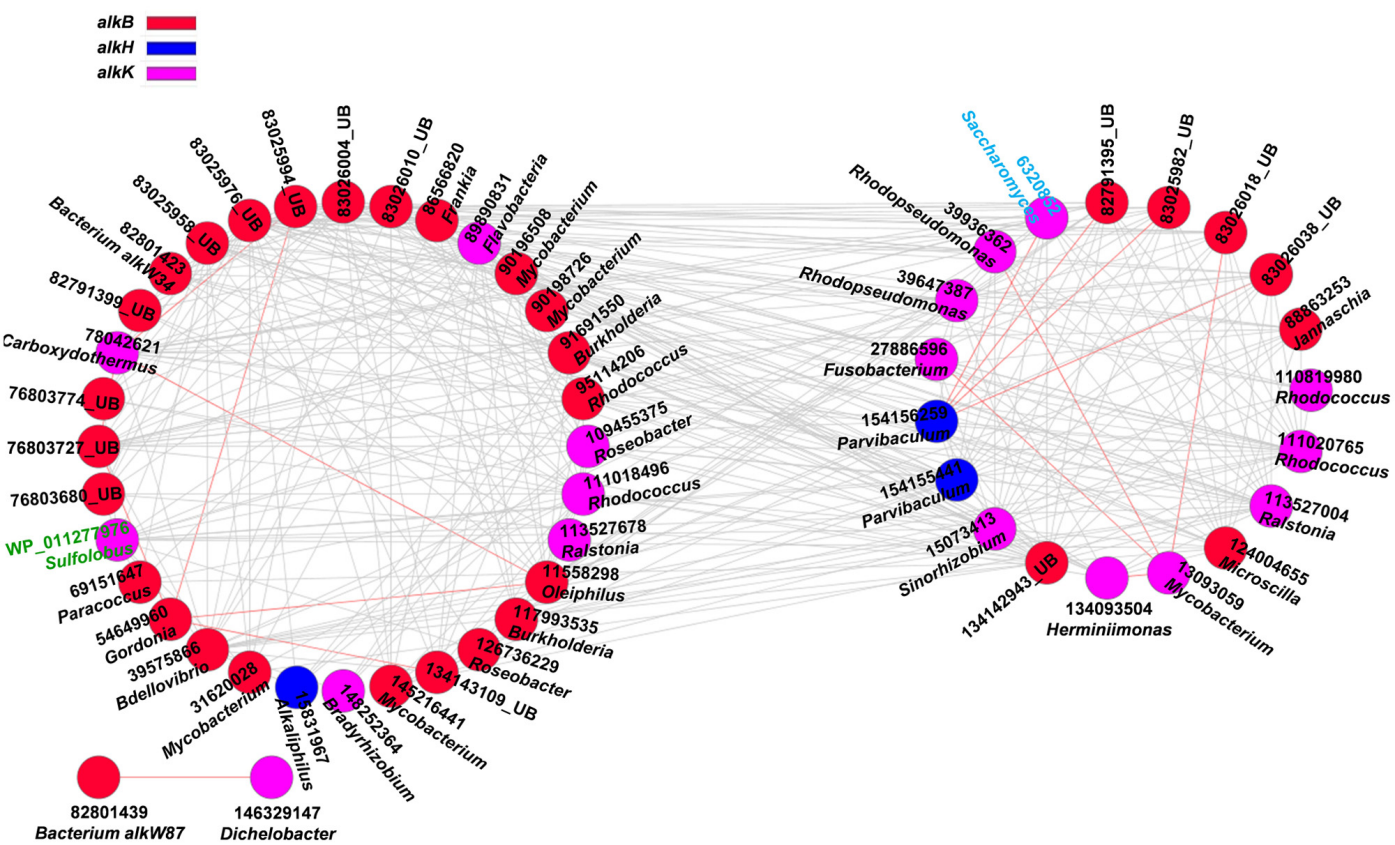
**Network interactions of the alk genes in the oil-contaminated soils.** The genes include *alkB* (alkane monooxygenase), *alkH* (aldehyde dehydrogenase), *alkJ* (alcohol dehydrogenase), and *alkK* (acyl-CoA synthetase). Numbers correspond to protein identification (National Center for Biotechnology Information, NCBI) and source genera. Black font indicates for bacteria (UB represents uncultured bacteria), green font for archaea, and blue font for fungi. Detailed information is listed in **Supplementary Table S1**. The two circles of the nodes represent different modules.

**FIGURE 3:**
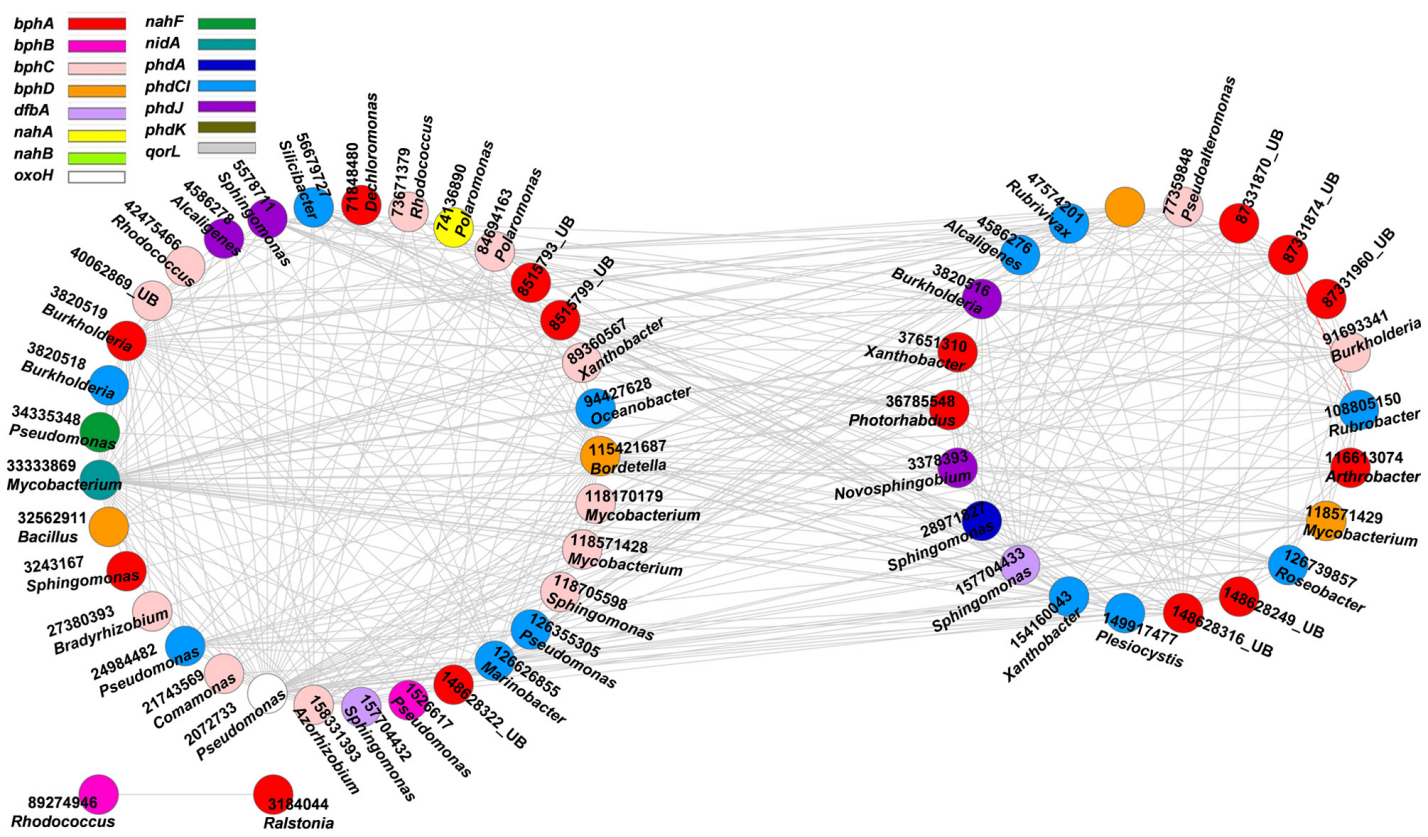
**Network interactions of the PAH genes in the oil-contaminated soils.** The genes are *bphA* (biphenyl 2,3-dioxygenase), *bphB* (*cis*-2,3-dihydrobiphenyl-2,3-diol dehydrogenase), *bphC* (biphenyl-2,3-diol 1,2-dioxygenase), *bphD* (2,6-dioxo-6-phenylhexa-3-enoate hydrolase), *nahA* (naphthalene 1,2-dioxygenase), *nahB* (*cis*-1,2-dihydro-1,2-dihydroxynaphthalene/dibenzothiophene dihydrodiol dehydrogenase), *nahF* (salicylaldehyde dehydrogenase), *phdA* (pyruvate dehydrogenase), *phdCI* (carboxylate isomerase), *phdG* (hydratase–aldolase), *phdJ* [4-(2-carboxyphenyl)-2-oxobut-3-enoate aldolase], *phdK* (2-carboxybenzaldehyde dehydrogenase), *aorL* (quinoline 2-oxidoreductase), *nidA* (putative ring-hydroxylating dioxygenase), *oxoH* (putative hydrolase). Numbers correspond to protein identification (NCBI) and source genera, all from bacteria (UB represents uncultured bacteria). Detailed information is listed in **Supplementary Table S2**. The two circles of the nodes represent different modules.

Aliphatic and aromatic hydrocarbons are major components of crude oil contaminants in oil-contaminated sites (Liang et al., 2012). To understand the co-occurrence levels of microbial functional genes involved in aliphatic and aromatic hydrocarbons degradation, we further constructed the networks of *alk* and PAH degradation genes in contaminated soils (**Figures 2** and **3**). **Supplementary Tables S1** and **S2** show detailed information on node degrees (links), gene identifications, names, and derived microorganisms.

Several functional genes, namely, *alkB*, *alkH*, and *alkK*, were detected in oil-contaminated soils. These genes were responsible for degrading aliphatic hydrocarbons. Overall, 95.4% of the total interactions of alkane-degrading genes were negative (**Figure 2**, **Supplementary Table S1**), which may reflect the competitive behavior of microbial functional communities in alkane degradation. The functional gene with the highest connectivities was *alkB* (83025976, uncultured bacterium) with 31 connections. All were negative, and the strength degree was 0.284 (clustering coefficient).

High concentrations of PAHs, such as naphthalene, phenanthrene, pyrene, chrysene, benzo(e)pyrene, and their alkylated derivatives, were detected in oil-contaminated sites (Cline et al., 2007). Thus, we further explored the co-occurrence of microbial functional gene involved in PAH degradation (**Figure 3**, **Supplementary Table S2**). All interactions of the PAH genes were negative. The functional gene with the highest connectivity was *nidA* (33333869, *Mycobacterium* sp.), with 47 connections and strength degree of 0.205.

### Association of Network Structure with Environmental Characteristics

Pearson correlation analysis was performed between gene degrees and environmental factors to determine the relationships among microbial network interactions, oil contamination and soil geochemical variables, (**Table 4**). Gene degree was calculated by summing the strengths of the connections (i.e., links) of each gene (i.e., node) with all of the other connected genes in the network. Gene degree represents how strong a gene is connected to other genes; this degree is one of the most commonly used network indices. Negative correlations (*P* < 0.01) were observed between gene degrees and oil concentration, and total nitrogen and total phosphorus in contaminated soils. This result indicates that these factors may reduce the co-occurrence of microbial functional genes in the community network because of the potential competitive relationships among several microbial groups for available carbon and nutrient sources. Some of the correlations were significant but with low correlation coefficient levels, thus indicating the weak effect of these factors to the gene degrees.

**Table 4:** Pearson correlations between gene degrees and environmental factors.

	Oil	Texture	TOC	TN	EN	TP	EP	pH	Water	Salt
BS-U	/	-0.10^∗∗^	-0.02	-0.08^∗^	-0.05	0.04	-0.04	-0.15^∗∗^	0.21^∗∗^	-0.18^∗∗^
BS-C	-0.19^∗∗^	0.04	-0.07	-0.25^∗∗^	0.08	-0.20^∗∗^	-0.07	-0.08	0.01	0.11


*^∗^Correlation is significant at 0.05 level (two-tailed). ^∗∗^Correlation is significant at 0.01 level (two-tailed). TOC, total organic carbon; TN, total nitrogen; EN, available nitrogen; TP, total phosphorus; EP, available phosphorus.*

## Discussion

To understand the influence of oil contamination on microbial interactions further, we investigated the changes of microbial molecular ecological networks in response to oil contamination. Network properties considerably changed in the contaminated samples when compared with the control. For example, connectivity, which provides information on how strong a node is connected to other nodes and is a commonly used network index (West, 1996), was reduced by 16.8% in response to oil contamination. Modularity measures the extent to which nodes possess more links within their own modules than expected if linkage is random. In this study, both module numbers and modularity decreased under oil contamination, with reductions of 76.6 and 41.8%, respectively. Small network sizes were also observed in contaminated soils than in the control at 256 and 754 functional genes (nodes) in the two networks, respectively. Although hydrocarbon contamination is known to exert profound effects on soil microbial community composition, diversity, and functional processes, the effects on the microbial ecological networks were first explored in this study. Findings revealed that the overall functional network structures were altered, thereby indicating a potential change in the organization of microbial communities (Faust and Raes, 2012). Mougi and Kondoh (2012) proposed that increasing complexity leads to increased stability in a community with mixed interaction types. Thus, the stability of microbial functional community may be reduced with the stress of oil contamination. Carbon input (as elevated CO_2_) has been reported to increase the complexity of soil microbial networks and produce a more stable community structure than usual (Zhou et al., 2010). Although hydrocarbons may have increased carbon source input and stimulated certain microbes that can utilize the carbons, degradable carbon was consumed and caused C:N imbalance. This imbalance may have resulted in the microbial functional instability. These findings can be partially confirmed by the decrease in microbial carbon substrate utilization accompanied by diversity loss in the culture-based Biolog analysis in the present study (**Supplementary Figure S1**). The insignificance of the distance-decay relationship (community similarity vs. geographic distance) in contaminated sites suggests that oil contamination significantly influences microbial communities to decrease in endemism, especially for the groups functioning in hydrocarbon degradation (Liang et al., 2015). Given that oil contamination cause a loss in the overall microbial diversity and alternation of community structures, many researchers concluded that anthropogenically induced oil contamination changed the microbial ecosystem (Hazen et al., 2010; Lu et al., 2012; Bell et al., 2013b). This change results in the potential switching of roles of microbial species and ecological functions of communities.

Identifying key populations/genes in a community is a challenge, because of the high diversity and uncultured status of microbes (Faust and Raes, 2012). In this study, fMEN analysis provided information on candidate genes/populations that are most important to microbial ecosystem structures and functions in oil-contaminated sites. We defined the two types of keystone genes. The first type refers to the genes that play key roles in the overall network based on network topology and their module memberships, such as module hubs (those highly connected to numerous genes in their own modules), connectors (those highly linked to several modules), and network hubs (acting as both module hubs and connectors). In this study, oil contamination changed the key genes in the ecological network. Two module hub genes (derived from *Nitrococcus* and *Nitrosomonas*, respectively) were present in the contaminated soils and 12 genes (mainly derived from *Rhodobacter*, *Geobacter*, *Xylella*, *Sphingopyxis*, etc.) in the uncontaminated soils with no overlap between the two conditions. Most of the module hub genes are functioning in carbon and nitrogen cycling, such as *pmoA*, *nifH*, and *nrfA*. The hub genes were derived from different organisms in the two conditions; hence, the changes of key module hubs by contamination may be due to the responses of microorganisms to the environmental stimulus. By contrast, 23 genes played as connectors (connecting modules) in the contaminated soils and none in the control. These genes mainly function in organic contaminant degradation, such as *alk*, *bco*, and *edbABC* (mainly derived from *Sphingomonas*, *Geobacillus*, *Novosphingobium*, *Trichoderma*, and *Deinococcus*), as well as *nifH* in nitrogen fixation. This result was expected because an increased number of genes and organisms functioning in hydrocarbon degradation were observed in oil-contaminated sites (Horvath, 1972; Baldwin et al., 2008; Bell et al., 2013b). We also observed that several genes played as module hub or connector function in other biogeochemical cycles, such as metal and antibiotic resistance. The actual roles of these hub or connector genes must be elucidated by real biological replicates of networks or co-culture experiments in the further work.

The second keystone genes are defined as those highly connected nodes (genes) involved in the degradation of the main components of oil contaminant. In the fMENs, patterns with a few highly connected nodes render the network more robust to change (Albert et al., 2000; Montoya et al., 2006). If highly connected nodes are lost, the network changes dramatically. Thus, these highly connected nodes may be analogous to microbial “keystone genes.” The top six keystone *alk* genes are *alkB* (83025976, 31 connections), *alkK* (89890831, 29 connections), *alkK* (111018496, 26 connections), *alkK* (39647387, 24 connections), *alkB* (134142943, 24 connections), and *alkH* (154155441, 24 connections). Many keystone genes in the *alk* gene network were derived from some species belonging to *Flavobacteria*, *Rhodococcus*, *Rhodopseudomonas*, and *Parvibaculum*. These bacteria are widely reported in alkane degradation (Naïtali et al., 2003; Mohanty and Mukherji, 2008). The top six keystone PAH genes are *nidA* (33333869, 47 connections), *oxoH* (2072733, 45 connections), *bphD* (115421687, 43 connections), *bphA* (3820519, 29 connections), *phdCI* (126626855, 26 connections), and *bphC* (84694163, 25 connections). These genes are mainly derived from *Mycobacterium*, *Pseudomonas*, *Bordetella, Burkholderia, Marinobacter*, and *Polaromonas*, which have been found to be capable of naphthalene and phenanthrene utilization (Chaillana et al., 2004), as well as the degradation of other petroleum hydrocarbons (Atlas, 1981; Hamamura et al., 2013; Meyneta et al., 2014). Our study further indicated that these functional groups carrying the keynote functional genes may play important roles in maintaining the stability of the biological network.

Microorganisms do not exist in isolation but form complex ecological interaction webs with several interaction types (Faust and Raes, 2012). Detecting and investigating various types of interactions in microbial ecosystems are difficult to accomplish (Raes et al., 2007), specifically in an environment disturbed by anthropogenic activities. A previous study experimentally demonstrated that increasing disturbance promotes microbial interspecies competition (Violle et al., 2010). Competition can constrain the specific functions of a community in several cases because of limited resources and habitat available to the most productive species (Bell et al., 2013a). In oil-contaminated soils, negative co-occurrence patterns prevailed among functional genes involved in alkane and PAH degradation. This finding suggests the competition for carbon compound and/or nutrient under oil contamination. For example, *Sphingomonas* is more competitive in nutrient acquisition than other genera in hydrocarbon-contaminated sites (Bell et al., 2011). In our study, we also observed that gene *xylJ* (28971837) derived from *Alphaproteobacteria* (*Sphingomonas* sp.) with the highest links in BS showed negative interactions with several other genes derived from *Gammaproteobacteria* (*Pseudomonas* sp. and *Aeromonas* sp.), *Actinobacteria* (*Mycobacterium* sp., *Nocardia* sp., and *Corynebacterium* sp.), *Spirochaetes* (*Spirochaeta* sp.), and *Firmicutes* (*Streptococcus* sp.). A small proportion of positive interactions, particularly, 4.5% of the total interactions in the *alk* functional gene network, were also observed. These interactions include those between *alkK* (6320852, *Saccharomyces*) and *alkH* (154156259, *Parvibaculum*); *alkB* (11558298, *Oleiphilus*) and the other two genes *alkB* (54649960, *Gordonia*) and *alkK* (78042621, *Carboxydothermus*); *alkK* (13093059, *Mycobacterium*) and the other four genes *alkK* (27886596, *Fusobacterium*), *alkK* (39936362, *Rhodopseudomonas*), *alkB* (83026018, uncultured bacterium), and *alkK* (134093504, *Herminiimonas*); and *alkB* (54649960, *Gordonia*) and the other three *alkB* genes (76803727, 83025994 and 134143109), which are all derived from uncultured bacterium. The positive interactions may reflect the commonly preferred environmental conditions or cooperative behaviors, such as cross feeding, syntrophic interactions, and mutualistic interactions (Raes and Bork, 2008; Steele et al., 2011).

Interactions between domains (bacteria, fungi, and archaea) were reported previously (Rousk et al., 2008; Steele et al., 2011; Bell et al., 2013b). In the current study, the network of functional genes involved in contaminant degradation showed that bacteria, fungi, and/or archaea were connected. Bacteria and fungi are generally described as antagonists for substrate competition in the soil environment (Mille-Lindblom et al., 2006; Meidute et al., 2008; Rousk et al., 2008). In oil-contaminated soils, the negative interactions among functional genes (*alk* and PAH degrading) derived from different microorganisms may be inferred as competition among microbial groups for degradable carbon sources, limited supply of nitrogen, and phosphorus.

Comprehensive information on microbial species or taxonomic units across relatively large numbers of samples is essential in detecting the co-occurrence relationships among microbial communities using network analysis (Barberan et al., 2012). Sample sets should ideally include spatial or temporal gradients in environmental conditions to achieve sufficient variability in taxon abundances to resolve co-occurrence patterns (Barberan et al., 2012). In the RMT-based molecular ecological network approach (Zhou et al., 2010), 10 samples are required to construct a network of microbial communities to ensure that the co-occurrence patterns are statistically significant rather than a random process. Thus, in this study, 20 soil samples (10 contaminated and 10 uncontaminated soils) were selected to infer the possible co-occurrence relationship between microbial functional genes under long-term oil contamination. Although we could not scale the results to all the situations with only a few samples, constructing a co-occurrence network is important to determine the potential interactions among different microorganisms. The results would provide better understanding of the responses of biological communities to severe environmental contamination. Additional sampling efforts combined with laboratory experiments are required to further obtain fundamental insight into microbial ecological networks in complex environmental habitats.

## Author Contributions

All authors contributed intellectual input and assistance to this study and manuscript preparation. GL, JZ, BS, and YL developed the original framework. YL and HZ contributed reagents and data analysis. YL, BS, and JZ wrote the paper.

## Conflict of Interest Statement

The authors declare that the research was conducted in the absence of any commercial or financial relationships that could be construed as a potential conflict of interest.

## Footnotes

1 http://ieg.ou.edu/microarray

2 http://ieg2.ou.edu/MENA
