# Supplementary material for: Long-Term Oil Contamination Alters the Molecular Ecological Networks of Soil Microbial Functional Genes
Source: Front Microbiol. 2016 Feb 3;7:60. doi: 10.3389/fmicb.2016.00060 (PMC4737900; doi:10.3389/fmicb.2016.00060)
Supplement: Supplementary file 1 [file Data_Sheet_1.DOCX]

Supplementary Material

Long-term oil contamination alters the molecular ecological networks of soil microbial functional genes

Yuting Liang^1*^, Xu Zhang^2^, Ye Deng^3^, Jizhong Zhou^2,4^, Guanghe Li^2^ and Bo Sun^1^

# Supplementary Figures and Tables

## Supplementary Figures

**Supplementary Figure 1.** Microbial metabolic activities that break down various carbon sources decrease significantly with long-term oil contamination based on the culture-based Biolog method.

## Supplementary Tables

**Supplementary Table S1.** Nodes information of the *alk* genes in oil contaminated soils from the BS site. The connectivity is defined as the links of the gene to all of the other genes in the network

| Gene id | Node connectivity | Gene name | Organism |
| --- | --- | --- | --- |
| 83025976 | 31 | *alkB* | uncultured bacterium |
| 89890831 | 29 | *alkK* | *Flavobacteria bacterium BBFL7* |
| 111018496 | 26 | *alkK* | *Rhodococcus sp. RHA1* |
| 39647387 | 24 | *alkK* | *Rhodopseudomonas palustris CGA009* |
| 134142943 | 24 | *alkB* | uncultured bacterium |
| 154155441 | 24 | *alkH* | *Parvibaculum lavamentivorans DS-1* |
| 111020765 | 23 | *alkK* | *Rhodococcus sp. RHA1* |
| 15073413 | 22 | *alkK* | *Sinorhizobium meliloti* |
| 113527004 | 21 | *alkK* | *Ralstonia eutropha H16* |
| 39575866 | 19 | *alkB* | *Bdellovibrio bacteriovorus HD100* |
| 90196508 | 19 | *alkB* | *Mycobacterium vanbaalenii PYR-1* |
| 91691550 | 19 | *alkB* | *Burkholderia xenovorans LB400* |
| 11558298 | 18 | *alkB* | *Oleiphilus messinensis* |
| 83025958 | 18 | *alkB* | uncultured bacterium |
| 83025994 | 18 | *alkB* | uncultured bacterium |
| WP_011277976 | 17 | *alkK* | *Sulfolobus acidocaldarius DSM 639* |
| 76803727 | 17 | *alkB* | uncultured bacterium |
| 78042621 | 17 | *alkK* | *Carboxydothermus hydrogenoformans Z-2901* |
| 95114206 | 17 | *alkB* | *Rhodococcus sp. SoF* |
| 109455375 | 17 | *alkK* | *Roseobacter denitrificans OCh 114* |
| 124004655 | 16 | *alkB* | *Microscilla marina ATCC 23134* |
| 154156259 | 16 | *alkH* | *Parvibaculum lavamentivorans DS-1* |
| 83026004 | 15 | *alkB* | uncultured bacterium |
| 83026010 | 15 | *alkB* | uncultured bacterium |
| 82791395 | 14 | *alkB* | uncultured bacterium |
| 6320852 | 13 | *alkK* | *Saccharomyces cerevisiae* |
| 83025982 | 13 | *alkB* | uncultured bacterium |
| 83026038 | 13 | *alkB* | uncultured bacterium |
| 27886596 | 12 | *alkK* | *Fusobacterium nucleatum subsp. vincentii ATCC 49256* |
| 39936362 | 12 | *alkK* | *Rhodopseudomonas palustris CGA009* |
| 83026018 | 12 | *alkB* | uncultured bacterium |
| 134093504 | 12 | *alkK* | *Herminiimonas arsenicoxydans* |
| 82801423 | 11 | *alkB* | *bacterium alkW34* |
| 88863253 | 11 | *alkB* | *Jannaschia sp. CCS1* |
| 134143109 | 10 | *alkB* | uncultured bacterium |
| 117993535 | 9 | *alkB* | *Burkholderia phytofirmans PsJN* |
| 145216441 | 9 | *alkB* | *Mycobacterium gilvum PYR-GCK* |
| 31620028 | 7 | *alkB* | *Mycobacterium bovis AF2122/97* |
| 76803680 | 7 | *alkB* | uncultured bacterium |
| 76803774 | 7 | *alkB* | uncultured bacterium |
| 86566820 | 6 | *alkB* | *Frankia sp. CcI3* |
| 110819980 | 6 | *alkK* | *Rhodococcus sp. RHA1* |
| 69151647 | 5 | *alkB* | *Paracoccus denitrificans PD1222* |
| 82791399 | 5 | *alkB* | uncultured bacterium |
| 158319675 | 5 | *alkH* | *Alkaliphilus oremlandii OhILAs* |
| 13093059 | 4 | *alkK* | *Mycobacterium leprae* |
| 54649960 | 4 | *alkB* | *Gordonia sp. TF6* |
| 148252364 | 4 | *alkK* | *Bradyrhizobium sp. BTAi1* |
| 126736229 | 3 | *alkB* | *Roseobacter sp. CCS2* |
| 82801439 | 1 | *alkB* | *bacterium alkW87* |
| 90198726 | 1 | *alkB* | *Mycobacterium vanbaalenii PYR-1* |
| 113527678 | 1 | *alkK* | *Ralstonia eutropha H16* |
| 146329147 | 1 | *alkK* | *Dichelobacter nodosus VCS1703A* |

**Supplementary Table S2.** Nodes information of the PAH genes in oil contaminated soils from the BS site. The connectivity is defined as the links of the gene to all of the other genes in the network

| Gene id | Node connectivity | Gene name | Organism |
| --- | --- | --- | --- |
| 33333869 | 47 | *nidA* | *Mycobacterium sp. S65* |
| 2072733 | 45 | *oxoH* | *Pseudomonas putida* |
| 115421687 | 43 | *bphD* | *Bordetella avium 197N* |
| 3820519 | 29 | *bphA* | *Burkholderia sp. RP007* |
| 126626855 | 26 | *phdCI* | *Marinobacter sp. ELB17* |
| 84694163 | 25 | *bphC* | *Polaromonas naphthalenivorans CJ2* |
| 8515799 | 22 | *bphA* | uncultured bacterium |
| 158331393 | 22 | *bphC* | *Azorhizobium caulinodans ORS 571* |
| 3378393 | 20 | *phdJ* | *Novosphingobium aromaticivorans* |
| 87331874 | 20 | *bphA* | uncultured bacterium |
| 148628316 | 20 | *bphA* | uncultured bacterium |
| 28971827 | 19 | *phdA* | *Sphingomonas sp. P2* |
| 40062869 | 19 | *bphC* | uncultured bacterium |
| 91693341 | 18 | *bphC* | *Burkholderia xenovorans LB400* |
| 108805150 | 18 | *phdCI* | *Rubrobacter xylanophilus DSM 9941* |
| 157704433 | 18 | *dfbA* | *Sphingomonas sp. LB126* |
| 5578711 | 16 | *phdJ* | *Sphingomonas xenophaga* |
| 24984482 | 16 | *phdCI* | *Pseudomonas putida KT2440* |
| 126739857 | 15 | *phdCI* | *Roseobacter sp. SK209-2-6* |
| 149917477 | 15 | *phdCI* | *Plesiocystis pacifica SIR-1* |
| 598361 | 14 | *bphD* | *Comamonas* |
| 36785548 | 14 | *bphA* | *Photorhabdus luminescens subsp. laumondii TTO1* |
| 37651310 | 14 | *bphA* | *Xanthobacter polyaromaticivorans* |
| 47574201 | 14 | *phdCI* | *Rubrivivax gelatinosus PM1* |
| 56679727 | 14 | *phdCI* | *Silicibacter pomeroyi DSS-3* |
| 77359848 | 14 | *bphC* | *Pseudoalteromonas haloplanktis TAC125* |
| 87331960 | 14 | *bphA* | uncultured bacterium |
| 118571429 | 14 | *bphD* | *Mycobacterium ulcerans Agy99* |
| 154160043 | 14 | *phdCI* | *Xanthobacter autotrophicus Py2* |
| 3820516 | 13 | *phdJ* | *Burkholderia sp. RP007* |
| 87331870 | 13 | *bphA* | uncultured bacterium |
| 116613074 | 13 | *bphA* | *Arthrobacter sp. FB24* |
| 148628249 | 12 | *bphA* | uncultured bacterium |
| 1526617 | 8 | *bphB* | *Pseudomonas putida* |
| 4586278 | 8 | *phdJ* | *Alcaligenes faecalis* |
| 21743569 | 8 | *bphC* | *Comamonas testosteroni* |
| 32562911 | 8 | *bphD* | *Bacillus sp. JF8* |
| 73671379 | 8 | *bphC* | *Rhodococcus erythropolis* |
| 94427628 | 8 | *phdCI* | *Oceanobacter sp. RED65* |
| 148628322 | 8 | *bphA* | uncultured bacterium |
| 4586276 | 6 | *phdCI* | *Alcaligenes faecalis* |
| 89360567 | 6 | *bphC* | *Xanthobacter autotrophicus Py2* |
| 3243167 | 4 | *bphA* | *Sphingomonas sp. CB3* |
| 3820518 | 4 | *phdCI* | *Burkholderia sp. RP007* |
| 8515793 | 4 | *bphA* | uncultured bacterium |
| 27380393 | 4 | *bphC* | *Bradyrhizobium japonicum USDA 110* |
| 34335348 | 4 | *nahF* | *Pseudomonas sp. ND6* |
| 71848480 | 4 | *bphA* | *Dechloromonas aromatica RCB* |
| 74136890 | 4 | *nahA* | *Polaromonas naphthalenivorans CJ2* |
| 118571428 | 4 | *bphC* | *Mycobacterium ulcerans Agy99* |
| 118705598 | 4 | *bphC* | *Sphingomonas wittichii RW1* |
| 126355305 | 4 | *phdCI* | *Pseudomonas putida GB-1* |
| 157704432 | 4 | *dfbA* | *Sphingomonas sp. LB126* |
| 3184044 | 1 | *bphA* | *Ralstonia sp. JS705* |
| 42475466 | 1 | *bphC* | *Rhodococcus rhodochrous* |
| 89274946 | 1 | *bphB* | *Rhodococcus sp. R04* |
| 118170179 | 1 | *bphC* | *Mycobacterium smegmatis str. MC2 155* |
